# Physiological health of wintering glaucous-winged gulls in coastal British Columbia

**DOI:** 10.1093/conphys/coaf048

**Published:** 2025-07-07

**Authors:** H Hall, M Hipfner, A Domalik, A Vanderpas, V Pattison, N Clyde, J Green, K A Hobson, T D Williams

**Affiliations:** Department of Biological Sciences, Centre for Wildlife Ecology, Simon Fraser University, 8888 University Drive West, Burnaby, BC V5A 1S6, Canada; Wildlife Research Division, Environment and Climate Change Canada, 5421 Roberston Road, Delta, BC VK4 3N2, Canada; Department of Biological Sciences, Centre for Wildlife Ecology, Simon Fraser University, 8888 University Drive West, Burnaby, BC V5A 1S6, Canada; Wildlife Research Division, Environment and Climate Change Canada, 5421 Roberston Road, Delta, BC VK4 3N2, Canada; Wildlife Research Division, Environment and Climate Change Canada, 5421 Roberston Road, Delta, BC VK4 3N2, Canada; Wildlife Research Division, Environment and Climate Change Canada, 5421 Roberston Road, Delta, BC VK4 3N2, Canada; Wildlife Research Division, Environment and Climate Change Canada, 5421 Roberston Road, Delta, BC VK4 3N2, Canada; Wildlife Research Division, Environment and Climate Change Canada, 5421 Roberston Road, Delta, BC VK4 3N2, Canada; Wildlife Research Division, Environment and Climate Change Canada, 5421 Roberston Road, Delta, BC VK4 3N2, Canada; Wildlife Research Division, Environment and Climate Change Canada,11 Innovation Blvd., Saskatoon, SK S7N 3H5, Canada; Department of Biology, University of Western Ontario, 1151 Richmond Street, London, ON N6A 3K7, Canada; Department of Biological Sciences, Centre for Wildlife Ecology, Simon Fraser University, 8888 University Drive West, Burnaby, BC V5A 1S6, Canada

**Keywords:** Anthropogenic impacts, biomarkers, biomonitor, seabirds, stable isotopes, wildlife health

## Abstract

Gulls (Laridae) use natural and urban environments and are useful ‘biomonitors’ of coastal ecosystem health. Here, we assessed physiological health of glaucous-winged gulls (*Larus glaucescens*, GWGU) wintering in the Salish Sea, British Columbia, Canada, a biodiverse region undergoing rapid anthropogenic change. We measured six physiological health biomarkers (blood glucose, triglycerides, haemoglobin, haematocrit, reactive oxygen metabolites and total antioxidants). Gulls sampled on the west coast of Vancouver Island had higher blood *δ*^13^C and *δ*^15^N values likely reflecting more marine diets compared with birds sampled in the Lower Mainland of Vancouver and in associated urban habitats such as landfills but terrestrial isotopic inputs are confounding. We found few differences in any of the six physiological markers in relation to region and habitat, or in overall indices of ‘health’ and ‘nutritional state’ using principal components analysis, even though these were characterized by varying levels of urban development and anthropogenic activity. Furthermore, individual variation in physiological traits was independent of individual variation in blood *δ*^13^C and *δ*^15^N values. This likely reflects the fact that we sampled ‘physiologically homeostatic’ individuals at all locations and habitats. Our study establishes reference values for six putative ‘health’ biomarkers, highlighting important covariates that need to be considered (e.g. sex, location) and provides a foundation for long-term physiological monitoring in relation to future anthropogenic impacts in this region.

## Introduction

During this era of rapid anthropogenically driven environmental change, marine ecosystems are under increasing stress from the impacts of coastal development (Bishop *et al.,* 2017), habitat degradation (Gibson *et al.,* 2007), overfishing (Jackson *et al.,* 2001), invasive species (Molnar *et al.,* 2008), ocean acidification (Cornwall and Eddy, 2015), climate change (Henson *et al.,* 2017) and pollution (Shahidul Islam and Tanaka, 2004). As such, understanding coastal ecosystem health is of vital importance (Tett *et al.,* 2013). Some of the very species that are most impacted by anthropogenic change can also act as useful ‘biomonitors’, providing insight into the state of ecosystems and emerging shifts that may be occurring (Piatt *et al.,* 2007; Mallory *et al.,* 2010). In this context, seabirds can be ideal biomonitors that can reflect changes in the marine environment through changes in their diet, habitat use, population trends, reproductive output and overall health (Moore and Kuletz, 2019). In particular, gulls (Family Laridae) are often used as indicators of marine and urban environmental health (e.g. Davis *et al.,* 2017; Laranjeiro *et al.,* 2020; Zorrozua *et al.,* 2020a) as they respond to anthropogenically modified habitats by utilizing both marine and terrestrial food subsidies (O’Hanlon *et al.,* 2020) and also utilize both natural and human-built structures for roosting and nesting (Kroc, 2018; Blight *et al.,* 2019).

Long-term population and diet monitoring of gulls has revealed changes in marine food webs over time (Blight *et al*., 2015; Hebert *et al.,* 1999). As generalist and opportunistic foragers, gulls can adapt to anthropogenic influence by foraging in landfills, agricultural land and urban areas (Bécares *et al.,* 2015; Juvaste *et al.,* 2017). Although access to anthropogenic subsidies can release gull populations from food constraints and increase overwinter survival (Duhem *et al.,* 2008; Zorrozua *et al.,* 2020b), in some cases it is also associated with negative health and reproductive consequences at both the individual and population level (Anderson *et al.,* 2019; Lopes *et al.,* 2022). In this context, conservation physiology (Madliger *et al.,* 2020) provides useful tools that can be utilized to evaluate underlying indicators of health in individuals and wildlife populations. Establishing reference values and conducting long-term physiological monitoring can provide insight into health issues before pathology is apparent (Dietz *et al.,* 2019), which helps to identify underlying mechanisms of population declines and increases the scope of environmental information gained from seabirds (Mallory *et al.,* 2010; Wells *et al.,* 2023). Although physiology can provide detailed insight into species- and ecosystem-level health, this tool is often overlooked and results in a lack of biomarker reference values for most species (Mallory *et al.,* 2010; Kophamel *et al.,* 2021). Additionally, studies of physiological status of non-breeding birds are even less frequent, despite the importance of health throughout the annual cycle (Mallory *et al.,* 2015; Minias, 2015; Merkling *et al.,* 2017).

The Salish Sea, on the northeastern edge of the Pacific Ocean of North America, encompasses the Strait of Georgia, the Strait of Juan de Fuca, British Columbia (BC) and Puget Sound, Washington (WA). This biologically productive area is a globally significant location for breeding and wintering birds (Crewe *et al.,* 2012; Gaydos *et al.,* 2015). Harbouring numerous human population centres, including three large cities (Vancouver and Victoria, BC and Seattle, WA), the Salish Sea is also strongly, and increasingly, influenced by human activity. In the marine environment, industrial fishing pressure, habitat degradation and the recovery of marine mammal populations have led to forage fish declines (Therriault *et al.,* 2009; Schweigert *et al.,* 2010), while legacy contaminants are an ongoing concern for both wildlife and humans (Chen *et al.,* 2012; Elliott and Elliott, 2016). Other local pollution threats include oil spills (Gaydos *et al.,* 2015), derelict fishing gear (Good *et al.,* 2009), vessel disturbance and the continued expansion of industrial development in estuarine habitats (Gaydos *et al.,* 2015). Currently, Environment and Climate Change Canada (ECCC) has a mandate to assess the health and habitat use of marine birds in this highly human-impacted area through the Salish Sea Marine Bird Monitoring and Conservation Program, in relation to the Trans-Mountain Pipeline expansion, which will increase crude oil tanker traffic in the Salish Sea by ~7-fold (Short, 2015). Canada also has a regional stewardship responsibility to migratory birds including glaucous-winged gulls (*Larus glaucescens*), which rely heavily on the Salish Sea region for breeding and overwintering (Environment Canada, 2013).

We assessed variation in physiological health, sex ratio and body mass of glaucous-winged gulls (GWGUs) wintering in the Salish Sea region of BC, in relation to anthropogenic activity (based on capture locations) and diet (via stable isotopes). In particular, we measured a suite of physiological markers commonly used in biomonitoring, which can be interpreted with respect to several aspects of health (Kophamel *et al.,* 2021; Whitehead and Dunphy, 2022). Plasma triglyceride and blood glucose levels provide insight into an individual’s ‘nutritional state’, with low values suggesting potential nutritional stress (Alonso-Alvarez and Ferrer, 2001). Likewise, haemoglobin and haematocrit levels are indicative of ‘aerobic capacity’ with poorer health seen in anaemic individuals resulting from factors such as poor nutrition, parasite loads and other aspects of condition (Minias, 2015). In relation to ‘oxidative status’, factors such as environmental conditions (Bodey *et al.,* 2019), contaminant exposure (King *et al.,* 2021), diet and exercise (Fowler and Williams, 2017; Fowler *et al.,* 2023; Monaghan *et al.,* 2009) can elevate concentrations of reactive oxygen metabolites (dROMs) in plasma, which lead to oxidative damage when an individual cannot adequately compensate by increasing their anti-oxidant capacity (‘OXY’; Costantini and Verhulst, 2009; Skrip and McWilliams, 2016). As such, we would consider birds with lower values for glucose, triglycerides, haematocrit, haemoglobin, or OXY (when high dROMs are present), to have poorer health. However, since interpretation of multiple biomarkers can be strengthened within the broader context of an individual’s health (Mallory *et al.,* 2010; Warne *et al.,* 2015; Whitehead and Dunphy, 2022) these six biomarkers were incorporated to generate indices of ‘overall health’ of individuals using principle components analysis. Specifically our objectives were to examine whether variation in a) each of these individual physiological biomarkers, or b) the overall health of individuals was explained by geographical region or habitat type (i.e. categorical variables) of capture sites, and c) in an exploratory approach, whether variation in physiological metrics was explained by (continuous) variation in diet (trophic level) or foraging habitat type (marine vs terrestrial) potentially indicated by variation in *δ*^15^N and *δ*^13^C values of gull blood cells. We predicted that a) physiological measures would vary with sampling location or habitat—given these had varying levels of urban development and anthropogenic activity—and b) that physiological measures would covary with any differences in stable isotope values among sampling locations.

## Materials and Methods

### Study area

**Figure 1:**
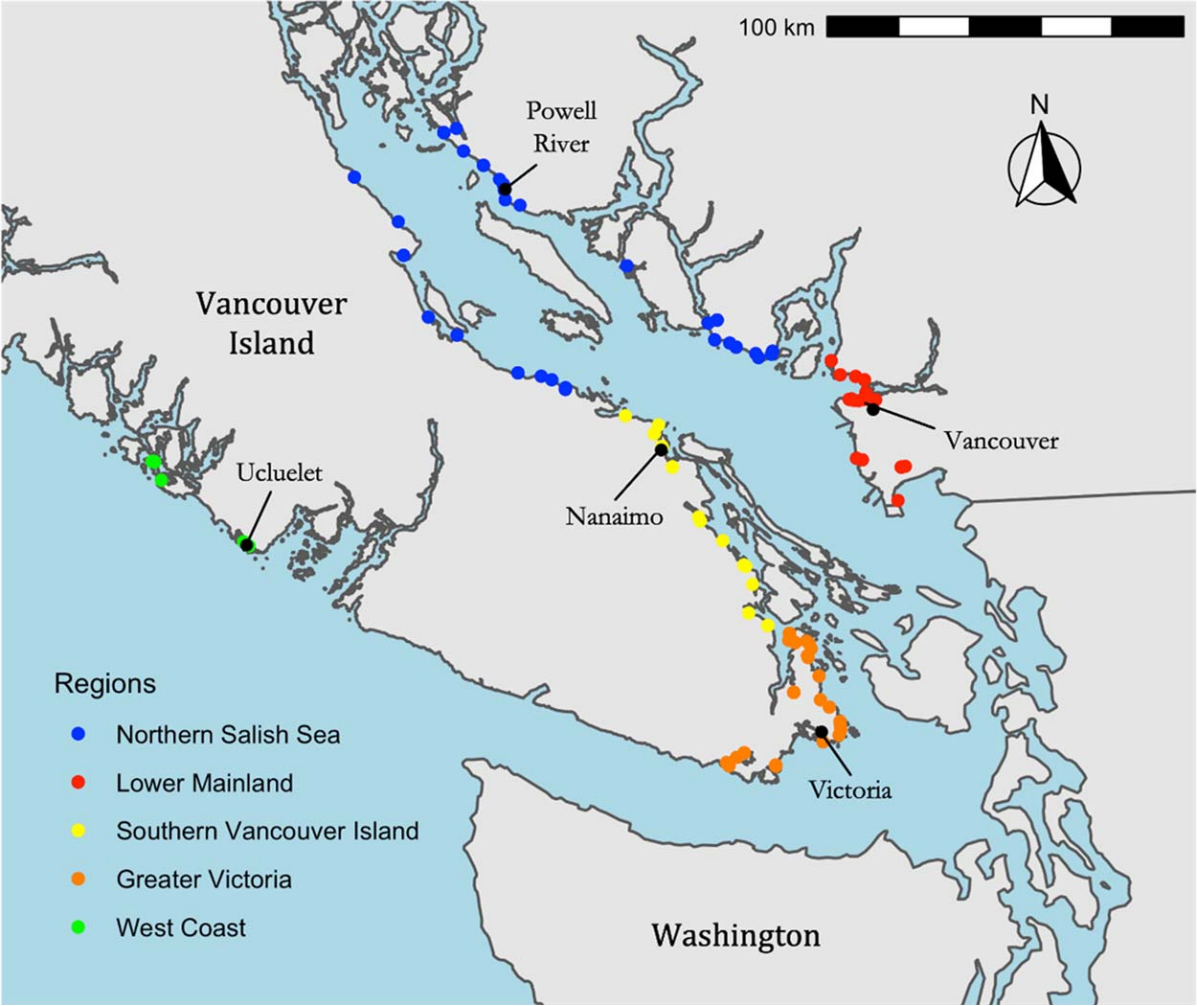
Adult GWGUs were captured and sampled among all four regions of the Salish Sea in both 2020 and 2021. Ten additional adults were sampled on the west coast of Vancouver Island in 2021 as an ‘outlier’ group for comparison.

The Salish Sea (49° 20′ 10.4”, -123° 50’ 21.6”) comprises two areas of sheltered marine waters harbouring large human population centres: the Strait of Georgia in Southern British Columbia and Puget Sound in Washington State, which are connected to the open Pacific Ocean by the exposed Strait of Juan de Fuca. In January and February of 2020 and 2021, we sampled adult GWGUs throughout the Canadian portion of the Salish Sea (Fig. 1). We attempted to sample evenly between the following regions: ‘Lower Mainland’, ‘Greater Victoria’, ‘Southern Vancouver Island’, and the ‘Northern Salish Sea’. Regions were categorized primarily by geographic proximity, but generally had similar beach substrate types and levels of anthropogenic influence throughout a given region (Supplemental Figs 1 and 2).

Within each region, we sampled as evenly as possible among various habitat types including landfills, ‘urban’ and ‘natural’ areas (Table 1). However, levels of human population density (Supplemental Fig. 1), urbanization and other types of land use varied among regions (Supplemental Fig. 2), therefore urban habitat types are over-represented in some regions (Table 1). For example, urban areas were comprised of beaches near high human population densities, as well as city parks, whereas ‘natural’ habitats included beaches in areas with considerably lower human population densities and less industrial activity. Gulls using landfills were also sampled in the Lower Mainland, Greater Victoria, Southern Vancouver Island and the Northern Salish Sea. Satellite imagery from the North American Land Change Monitoring System database was used to guide categorization of capture locations into ‘urban’ versus ‘natural’ habitat types (250 × 250 m resolution, North American Land Change Monitoring System, 2021) while human population density (people/km^2^) was obtained using census data (Statistics Canada, 2017). All three habitat types and all four regions of the Salish Sea were sampled in both years of study. Additionally, in 2021, 10 gulls were sampled on the west coast of Vancouver Island near the small towns of Ucluelet and Tofino as an ‘outlier’ group for comparison with Salish Sea birds.

**Table 1:** Distribution of GWGU capture location habitat types within each sampling region

	Landfill	Urban	Natural	West Coast	Total
Lower Mainland	8	26	0	0	34
Greater Victoria	10	36	9	0	55
Southern Vancouver Island	7	24	5	0	36
Northern Salish Sea	8	37	23	0	68
West Coast	0	0	0	10	10
Total	33	123	37	10	

Habitat types of capture locations were not differentiated for the gulls caught in the outgroup sampling area, the west coast of Vancouver Island (*n* = 10). All values reported are sample sizes. All habitats and regions were sampled in both 2020 and 2021, except for the ‘west coast’, which was not sampled in 2020.

### Data collection

Adult gulls were live-captured primarily using baited noose mats (Liu *et al.,* 2017) and occasionally with pneumatic CO_2_ net guns, when bait was not an effective attractant (Edwards and Gilchrist, 2011). As soon as possible after capture, we collected no more than 6 ml of blood (<1% of body weight) from the brachial vein of one or both wings using a 27.5-gauge heparinized needle and syringe. We also collected ~20 μl of whole blood from tarsal veins using a non-heparinized lancet and capillary tube. Of this, half was stored in 95% ethanol for molecular sexing, and the rest was used to measure glucose levels (mmol/l) in the field using a handheld glucose meter (Accu-check Aviva; Roche, Basel, Switzerland). In the field, samples for haemoglobin analysis were prepared by adding 5 ml of fresh, whole blood to 1.25 ml of Drabkin’s reagent (D5941 Sigma-Aldrich Canada, Oakville, Ontario, Canada). Haematocrit was measured on two capillary tubes that were immediately filled with fresh, whole blood and stored at 4°C for up to 6 h before centrifuging for three min at 13 000 g (Microspin 24; Vulcon Technologies, Grandview, Missouri, USA). All remaining blood was stored in a heparinized vacutainer at 4°C for up to 6 h before being centrifuged for 10 min at 5000 rpm. Plasma (without lipid extraction) and red blood cells were separated and stored at −20°C for up to 1 month and then at −80°C until assayed. Handling time was calculated for each individual and defined as the number of minutes from capture time until blood collection was finished. Before release, all gulls were photographed, aged, banded and morphometric measurements were collected including mass (± 20 g) and tarsus length (± 1 mm).

All research was conducted under ECCC Banding Permit #10667F, and ECCC Migratory Bird Sanctuary Permit #MM-BC-2020-0002. Animal use protocols were approved by ECCC’s Western and Northern Animal Care Committee (21MH03), as well as the Simon Fraser University Animal Care Committee (protocol no. 1318B-20). All personnel completed mandatory Animal Care training.

### Plasma analysis

Plasma triglyceride levels (mmol l^−1^) were analysed with a colorimetric assay according to the manufacturer’s instructions (Sigma-Aldrich Co.; also see Fowler and Williams, 2017). Haematocrit was measured using digital callipers (± 0.1 mm) and determined as a percentage of packed red cell volume to total column height (plasma plus packed red cell volume). Haemoglobin was measured using the cyanomethemoglobin method (Drabkin and Austin, 1932) modified for use with a microplate spectrophotometer and absorbance read at 540 nm (Wagner *et al.,* 2008). Total antioxidant titres (OXY; μmol HClO ml^−1^) and reactive oxygen metabolites (dROMs; mg H_2_O_2_ dl^−1^) in the plasma were measured using OXY and dROMs kits, respectively, from Diacron International (Grosseto, Italy). OXY absorbances were read at 490 nm, and dROMs at 546 nm, using protocols modified after Guindre-Parker *et al.* (2013) and Casagrande *et al.* (2012), respectively. All assays were run using 96-well plates and a microplate spectrophotometer (BioTek Powerwave 340; BioTek Instruments, Winooski, Vermont). For quality control, samples with an intra-assay coefficient of variation (CV) > 10% when assayed in triplicate (haemoglobin and OXY), or CV > 12% if run in duplicate (triglycerides and dROMs), were re-assayed if no obvious outlier could be removed. Inter-assay variation was 4.11% (triglycerides), 2.20% (haemoglobin), 9.41% (OXY), 10.57% (dROMs), and intra-assay variation was 6.74% (triglycerides), 1.60% (haemoglobin), 4.27% (OXY), and 6.69% (dROMs).

### Sexing

To determine gull sex, DNA was extracted from blood stored in 95% ethanol using a modified Chelex protocol (Burg and Croxall, 2001; Walsh *et al.,* 2013). Individuals were sexed using the Z43BF/Z43BR Primer Pair (Dawson *et al*., 2016); the forward primer was modified with M13 to allow incorporation of fluorescent marker to run on Licor gel. All polymerase chain reactions (PCRs) were conducted in 10 μl reactions with 1 μl of genomic DNA. PCR cocktails contained 2.0 μl ClearFlexi Buffer 5x (Promega), 2.5 mM MgCl₂, 200 μM dNTP, 1 μM each primer, 0.05 μM M13 primer and 0.5 units GoTaq (Promega). We used the following Thermocycler Conditions: 1 cycle of 30 s at 94°C; 35 cycles of 30 s at 94°C, 45 s at 55°C and 45 s at 72°C, with a final extension for 5 min at 72°C, and 5 s at 4°C. All PCR products were run on a 6% acrylamide gel. All gels included known positives (one male and one female) to maintain consistency across gels.

### Stable isotopes

For carbon and nitrogen stable isotope analyses, we weighed 1 mg of freeze-dried whole blood into pre-combusted tin capsules. Encapsulated blood was combusted at 1030°C in a Carlo Erba NA1500 or Eurovector 3000 elemental analyser. The resulting N_2_ and CO_2_ were separated chromatographically and introduced to an Elementar Isoprime (Elementar; Langenselbold, Germany) or a Nu Instruments Horizon (Nu Instruments Ltd; Wrexham, UK) isotope ratio mass spectrometer. We used two reference materials to normalize the results to VPDB and AIR: BWBIII keratin (*δ*^13^C = −20.18‰, *δ*^15^N = +14.31‰, respectively) and PRCgel (*δ*^13^C = −13.64‰, *δ*^15^N = +5.07‰, respectively). Within-run (*n* = 5) precisions as determined from both reference and sample duplicate analyses were ±0.1‰ for both *δ*^13^C and *δ*^15^N.

### Data analysis

For all physiological biomarkers measured, sample distributions were examined for normality and whether values were biologically plausible based on reference values for other gulls (e.g. Newman *et al.,* 1997; Minias, 2015; Laranjeiro *et al.,* 2020). Based on these reference values, biologically implausible outliers were removed for haemoglobin (*n* = 6; >24 g/dl) and OXY (*n* = 1; <110 μmol HClO/ml). Log transformations were used for triglycerides, glucose and dROMs (Fowler and Williams, 2017). Statistical analyses were performed, with significance determined using an alpha level of 0.05, in *R version 4.3.3* (R Core Team, 2024). Pearson’s correlation coefficients were used to examine pairwise relationships between the six physiological biomarkers measured and to test the potential effect of handling time on each trait. To address potential bias due to sex differences in our data, we first determined whether the ratio of females to males sampled was significantly different a) among capture years and b) between years, using the Chi-squared test. We also tested whether mass significantly varied with gull sex using Analysis of Variance (ANOVA).

Using linear mixed-effects models, we determined whether variation in any of the six biomarkers measured was explained by a) sex, b) mass, c) sex + mass or d) sex*mass. Free fatty acids in plasma can impact dROMs assay results (Pérez-Rodríguez *et al.,* 2015), so we additionally tested whether triglycerides, or any combination of triglycerides, sex and mass explained significant variation in dROMs measurements. All models were run with year as a random effect, except for haemoglobin, which was only measured in 2021. We used Akaike Information Criterion for small sample sizes (AICc) to determine the model of best fit using the MuMIn package (*version 1.48.4*; Bartoń, 2024). For a given trait, if the model with the lowest AICc score included mass as a significant effect, it was treated as a covariate, while a significant effect of sex was instead included as an interaction term with region or habitat in future models. This was to account for a likely skewed sex-ratio among regions and habitats, which could not be formally assessed due to uneven sample sizes. If neither mass nor sex were significant, no covariate or interaction term was included.

Next, we assessed whether each trait varied significantly by a) region or b) habitat type at capture using ANOVA (*lme4 version 1.1–35.5*; Bates *et al.,* 2015) and *post hoc* Tukey tests for pairwise comparisons. Least-squares means were calculated using the *emmeans* package (*version 1.10.4*; Lenth, 2024). To control for potential environmental variation between sampling years, year was included as a random effect for all models, excluding haemoglobin, which was only measured in 2021. Covariates (mass and/or triglycerides) and interaction terms (sex*region or sex*habitat and sex*mass) determined previously by model selection (described above) were included as needed.

Principal components analysis (PCA) was used to examine the pattern of correlations and distributions among GWGU physiological biomarkers, and to provide indices of overall ‘health’ for gulls wintering in the Salish Sea. Using the same approach as described above for individual biomarkers, we determined if any significant covariates (i.e. mass and/or sex) should be controlled for with PC variables. Additionally, PCA scores for individuals were used to compare physiological health among regions and habitat types of capture locations (R Core Team, 2024). Specifically, we tested whether individual scores from the first principal component (PC1) or the second principal component (PC2) varied significantly among region or habitat type using ANOVA and *post hoc* Tukey tests for pairwise comparisons.

For stable isotope data, we also determined whether to control for covariation due to sex and/or mass, and then tested if a) *δ*^13^C and *δ*^15^N varied among sampled regions or habitats using ANOVAs with *post hoc* Tukey tests, and then b) if individual variation in *δ*^13^C and *δ*^15^N were correlated with each of the six physiological biomarkers we measured, as well as the two PCA-derived variables of overall ‘health’*.*

## Results

A total of 202 adult GWGUs were sampled in 2020 (*n* = 53) and 2021 (*n* = 149). Body mass and all physiological biomarkers were independent of handling time (i.e. time between initial capture and collection, Table 2; *P* > 0.05 in all cases). Mass was negatively correlated with haemoglobin and with dROMs (Table 2; *P* > 0.05 in both cases). Haematocrit and glucose were also negatively correlated (r = −0.287, *P* < 0.001), and several pairwise comparisons of biomarkers demonstrated positive correlations, including: triglycerides and glucose, haemoglobin and haematocrit, OXY and dROMs, haematocrit and dROMs and triglycerides and dROMs (Table 2; all *P* < 0.05).

**Table 2:** Correlation coefficients (*r*) for physiological variables, handling time and body mass measured for GWGUs captured in southern British Columbia, Canada

	Trig. (mmol/l)	Gluc. (mmol/l)	Hb (g/dl)	PCV (%)	OXY (μmol HClO/ml)	dROMs (mg H_2_O_2_/dl)
Body mass (g)	−0.135	0.011	−***0.203***	0.035	−0.001	−***0.167***
Triglycerides		***0.206***	0.033	−0.027	0.129	***0.246***
Glucose			−0.004	−***0.287***	−0.049	−0.012
Haemoglobin (Hb)				***0.292***	0.064	0.064
Haematocrit (PCV)					0.099	***0.282***
Total antioxidants (OXY)						***0.199***
Handling time (s)	0.037	0.186	−0.111	0.170	0.021	0.047

Triglyceride, glucose and dROM variables were log transformed. Significant correlation coefficients are in italics/bold = *P* < 0.05.

Sex was determined for 197 out of 202 birds sampled. The sex ratio of our sample of adult gulls was significantly skewed (χ^2^ = 28.6; *df* = 1; *P* < 0.001), with more females (69.0%; *n* = 136) sampled than males (31.0%; *n* = 61). The sex ratio also differed significantly between capture years (χ^2^ = 9.57; *df* = 1; *P* < 0.002) with females representing 86.3% of birds captured in 2020 (44/51) and 63.0% in 2021 (92/146). Body mass varied significantly by sex (F_1,192_ = 207.4; *P* < 0.001) with males (1173 ± 123 g) weighing more on average than females (951 ± 86 g).

**Figure 2:**
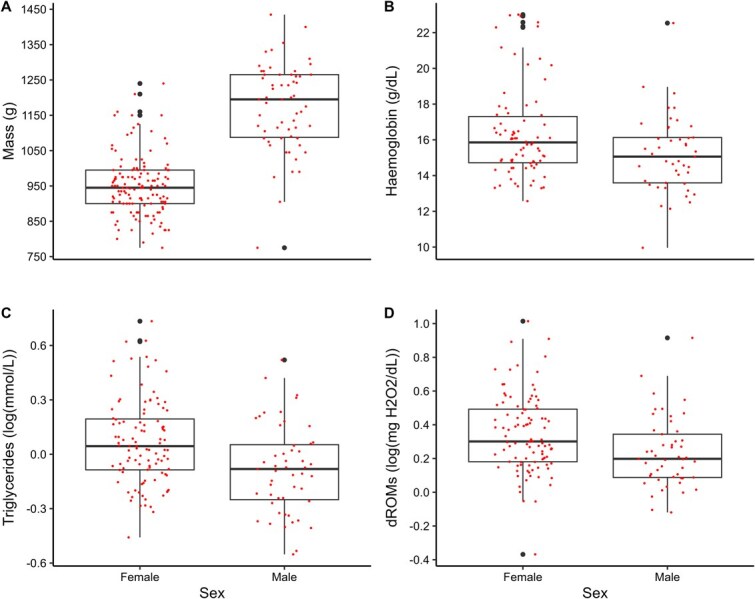
Adult female GWGUs weighed less than males, but had significantly higher haemoglobin, triglyceride and dROM levels in their blood (*P* < 0.05 in all cases). Note that triglycerides and dROMs values were log transformed.

Plasma triglyceride levels also varied with sex (Fig. 2), being higher in females (0.07 ± 0.23 log(mmol l^−1^)) than males (−0.08 ± 0.24 log(mmol l^−1^)); therefore sex was included as an interaction term in ANOVAs testing variation of mass or triglycerides by region or habitat (Supplemental Table 1). Likewise, haemoglobin was significantly higher in females (16.4 ± 2.59 g/dl) than males (15.2 ± 2.16 g/dl; Fig. 2) and was the only biomarker that varied with mass (F_3,114_ = −0.009; *P* = 0.03). The best model for testing variation in haemoglobin included sex, mass and a significant interaction between sex and mass (Supplemental Table 1). dROM levels were significantly higher in the plasma of female gulls (2.56 ± 1.59 log(mg H_2_O_2_ dl^−1^)) than in male gulls (1.95 ± 1.25 log(mg H_2_O_2_ dl^−1^); Fig. 2) and varied significantly triglycerides levels (Supplemental Table 1), However, the model with only triglycerides as a covariate produced the lowest AICc score; therefore we included triglycerides as a covariate in ANOVAs testing the variation of dROMs levels by region or habitat type of capture locations (Supplemental Table 1). Haematocrit, OXY and glucose levels did not vary with sex or mass.

### Variation in body mass and physiological traits by region

Body mass, triglycerides, glucose, haemoglobin and OXY levels were all independent of region (Table 3, *P* > 0.07 in all cases). Haematocrit (packed cell volume; PCV%) varied significantly among regions (F_4,142_ = 5.8; *P* < 0.001): birds from Greater Victoria had significantly higher haematocrit than birds captured in the Northern Salish Sea and Lower Mainland (*P* < 0.001 in both cases); no other pairwise comparisons among regions were significant (Table 3; *P* > 0.17 in all cases). There was a marginally non-significant effect of region on dROMs (F_4,143_ = 2.09; *P* = 0.085) with birds in the Greater Victoria region having higher dROMs levels than birds captured in the Northern Salish Sea (*P* < 0.08). All other pairwise comparisons of dROMs among regions were non-significant (Table 3; *P* > 0.4 in all cases).

**Table 3:** Variation in physiological variables by region (site of capture)

Trait	West Coast	Northern Salish Sea	Southern Vancouver Island	Greater Victoria	Lower Mainland
Body mass (g)	1068 ± 38 (9)	1048 ± 19 (67)	1099 ± 22 (33)	1049 ± 15 (53)	1072 ± 22 (32)
Triglycerides (mmol/l)	−0.04 ± 0.10 (9)	−0.03 ± 0.06 (53)	0.02 ± 0.06 (29)	0.02 ± 0.04 (38)	−0.02 ± 0.07 (25)
Glucose (mmol/l)	1.2 ± 0.1 (9)	1.2 ± 0.1 (52)	1.2 ± 0.1 (30)	1.2 ± 0.1 (36)	1.2 ± 0.1 (24)
Haemoglobin (Hb; g/dl)	15.3 ± 0.9 (9)	15.2 ± 0.5 (41)	14.4 ± 0.6 (23)	14.6 ± 0.6 (25)	15.6 ± 0.6 (23)
Haematocrit (PCV %)	43.7 ± 2.7 (9)	43.6 ± 2.4 (50)	45.6 ± 2.5 (30)	46.9 ± 2.4 (36)	42.9 ± 2.5 (25)
Total antioxidants (OXY; μmol HClO/ml)	239 ± 14 (9)	234 ± 8 (44)	243 ± 9 (26)	254 ± 6 (34)	240 ± 10 (26)
Reactive oxygen metabolites (dROMs; mg H_2_O_2_/dl)	0.34 ± 0.08 (10)	0.28 ± 0.05 (50)	0.37 ± 0.06 (30)	0.41 ± 0.05 (38)	0.30 ± 0.07 (23)

Values are least square means ± standard error values with sample sizes in parentheses. See text for details of analysis.

Note: Triglycerides, glucose and dROMs were log transformed. Models for all traits were run with year as a random effect, except for haemoglobin, which was only sampled in 2020. Models for mass, triglycerides and haemoglobin included sex as an interaction term. Sex*mass was included as an additional interaction term for haemoglobin and the model for dROMs included triglycerides as a covariate.

PCA was conducted on a subset of data, including only individuals for which all six physiological biomarkers were measured (*n* = 90). PC1 explained 27.0% of the variation among the physiological variables, and PC2 explained 24.3% of total variation. PC1 was positively influenced by dROMs, haematocrit, haemoglobin, OXY and weakly by triglycerides, while glucose had a similarly weak but negative influence (Supplemental Table 2). Conversely, PC2 was positively influenced by triglycerides, glucose, OXY and dROMs, but negatively by haematocrit and haemoglobin (Supplemental Table 2). When testing for covariation of sex and/or mass, only PC1 varied significantly with sex (Supplemental Table 1), which was included as an interaction term with models testing variation in PC1 by region and habitat. Neither PC1, a putative measure of overall health, or PC2, a putative measure of nutritional status, varied among regions (F_4_ = 1.10; *P* > 0.3; F_4_ = 0.95; *P* > 0.4, respectively).

### Variation in body mass and physiological traits by habitat type

Body mass differed significantly with habitat type of capture location (F_3,186_ = 2.80; *P* = 0.04, controlling for sex and sex*habitat type): birds captured at landfills had higher body mass than those captured in natural habitats (Table 4; *P* < 0.04). No other pairwise comparisons of body mass among habitat types were significant (*P* > 0.2 in all cases). Haemoglobin levels varied with the habitat type of capture locations (F_3,111_ = 3.68; *P* < 0.02): haemoglobin levels were higher in birds caught at landfills compared to urban sites (Table 4; *P* = 0.05), but all other pairwise contrasts were non-significant (*P* > 0.5 in all cases). All other physiological traits were independent of habitat type (Table 4; *P* > 0.30). Similarly, neither PC1 (F_3_ = 1.55; *P* = 0.21) or PC2 (F_3_ = 0.82; *P* = 0.5), from PCA, differed significantly among habitats.

**Table 4:** Variation in physiological variables by habitat type (site of capture)

Trait	West Coast	Natural	Urban	Landfill
Body mass (g)	1068 ± 38 (9)	1022 ± 23 (36)	1061 ± 12 (118)	1103 ± 23 (31)
Triglycerides (mmol/l)	−0.037 ± 0.099 (9)	0.021 ± 0.062 (34)	−0.024 ± 0.041 (90)	0.029 ± 0.073 (21)
Glucose (mmol/l)	1.2 ± 0.1 (9)	1.2 ± 0.1 (30)	1.2 ± 0.04 (90)	1.2 ± 0.1 (22)
Haemoglobin (Hb; g/dl)	15.9 ± 0.9 (9)	15.8 ± 0.7 (24)	14.8 ± 0.4 (90)	16.5 ± 0.7 (20)
Haematocrit (PCV %)	44.3 ± 2.3 (9)	44.9 ± 1.9 (31)	45.4 ± 1.8 (89)	44.7 ± 2.0 (21)
Total antioxidants (OXY; μmol HClO/ml)	239 ± 14 (9)	238 ± 8 (26)	246 ± 6 (84)	231 ± 10 (20)
Reactive oxygen metabolites (dROMs; mg H_2_O_2_/dl)	0.349 ± 0.09 (10)	0.369 ± 0.06 (31)	0.347 ± 0.06 (88)	0.270 ± 0.07 (22)

Values are least square means ± standard error values with sample sizes in parentheses. See text for details of analysis.

Note: Triglycerides, glucose and dROMs were log transformed. Models for all traits were run with year as a random effect, except for haemoglobin, which was only sampled in 2020. Models for mass, triglycerides and haemoglobin included sex as an interaction term. Sex*mass was included as an additional interaction term for haemoglobin and the model for dROMs included triglycerides as a covariate.

### Variation in ***δ***  ^**13**^**C and**  ***δ***  ^**15**^N in relation to physiological health, region and habitat

**Figure 3:**
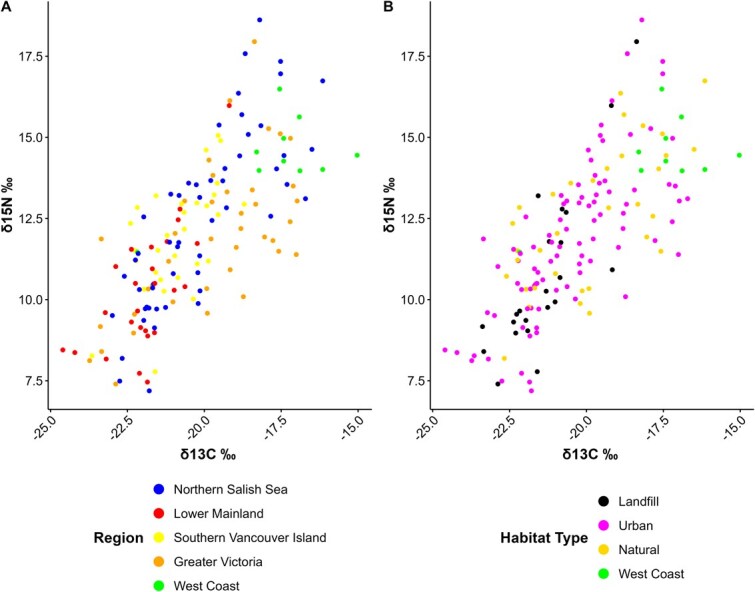
The strong and positive relationship between *δ*^13^C and *δ*^15^N ‰ stable isotope values, measured in red blood cell fractions from adult GWGUs, was demonstrated throughout regions (A) and habitat types (B) of sampling locations.

**Figure 4:**
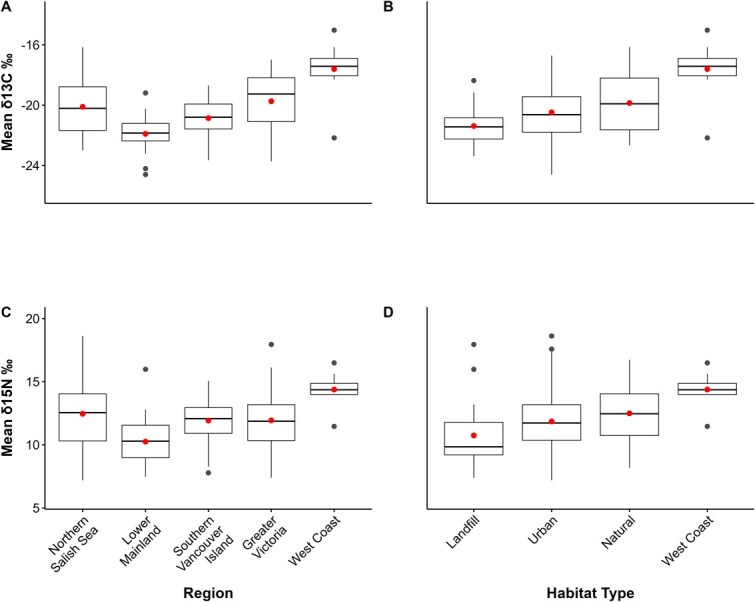
Variation in *δ*^13^C (A and B) and *δ*^15^N (C and D) in relation to region (A and C) and habitat type (B and D) of adult GWGU capture locations. Dots in the center of boxes indicate mean stable isotope values and boxes with different lowercase letters are significantly different (*P* < 0.05) using ANOVA and Tukey *post hoc* pairwise comparisons.

*δ*  ^13^C and *δ*^15^N measured in GWGU blood samples had a strong, positive correlation (r = 0.74, *P* < 0.001) and were continuously distributed in relation to region and habitat type (Fig. 3). However, individual variation in mass, all physiological traits and PC1 and PC2 were independent of variation in both blood *δ*^13^C and *δ*^15^N (Table 5; *P* > 0.05 in all cases) and neither stable isotope had significant covariation with mass or sex (Supplemental Table 1). In contrast, both mean *δ*^13^C (F_4,160.4_ = 14.12; *P* < 0.001) and mean *δ*^15^N (F_4,144.1_ = 7.19; *P* < 0.001) varied by region (including year as a random factor). Similarly, both mean *δ*^13^C (F_3,108.7_ = 11.53; *P* < 0.001) and mean *δ*^15^N (F_3,101.6_ = 6.44; *P* < 0.001) varied among habitat types (Fig. 4).

**Table 5:** Correlation coefficients (*r*) demonstrate no relationships with either *δ*^13^C ‰ or *δ*^15^N ‰ stable isotope values in pairwise comparisons with mass, physiological traits and principal components 1 and 2 (PC1 and PC2) measured in adult GWGUs wintering in southern British Columbia, Canada

Trait	*δ* ^13^C ‰	*δ* ^15^N ‰
Body mass (g)	−0.031	−0.049
Triglyceride (mmol/l)	−0.121	−0.098
Glucose (mmol/l)	−0.111	−0.058
Haemoglobin (g/dl)	−0.024	0.083
Haematocrit (PCV %)	0.037	0.077
Total antioxidant titres (OXY; μmol HClO/ml)	0.101	−0.031
Reactive oxygen metabolites (dROMs; mg H_2_O_2_/dl)	0.070	0.045
PC1	0.0056	0.0105
PC2	−0.144	−0.086

Note that triglyceride, glucose and dROM values were log transformed. Values in italics/bold = *P* < 0.05.

GWGUs sampled on the west coast of Vancouver Island had significantly higher *δ*^13^C values compared to all other regions (*P* < 0.02 in all cases) with the lowest *δ*^13^C values in birds sampled in the Lower Mainland (Fig. 4A). Birds from the Northern Salish Sea also had higher *δ*^13^C values than Lower Mainland birds (*P* < 0.01; Fig. 4A). Similarly, birds sampled on the west coast of Vancouver Island and the Northern Salish Sea had higher *δ*^15^N than Lower Mainland birds (*P* < 0.001 in both cases; Fig. 4C). For habitat type, gulls captured on the west coast and at natural habitats had higher *δ*^13^C (Fig. 3B) and higher *δ*^15^N (Fig. 4D) values compared with birds caught at landfills (*P* < 0.25 in all cases).

## Discussion

We used a physiological approach to assess the health of a key seabird indicator species (Barry, 2015), the GWGU, wintering in the biologically important coastal Salish Sea ecosystem. Stable isotope analysis of GWGU red blood cells confirmed differences among sampling regions and habitat types, likely reflecting differences in diet and the relative proportions of marine versus terrestrial foods, and demonstrated continuous variation among individuals (Blight *et al.,* 2014; Hobson *et al.,* 2015). However, individual variation in physiological traits was independent of individual variation in *δ*^13^C and *δ*^15^N values, and likewise, we found very few differences in physiological markers in relation to region and habitat, even though these were characterized by varying levels of urban development, anthropogenic activity and proximity to marine foods. Although there was significant covariation among several of the six measured physiological traits, we found that principal component scores, which combined the multiple traits into unified measures of overall ‘health’ or ‘nutritional status’, were also independent of sampling region and habitat. Nevertheless, our study has established reference values for six putative ‘health’ biomarkers, highlighting important covariates that need to be considered (e.g. sex), and provides a foundation for long-term physiological monitoring in relation to future anthropogenic impacts in this region. The similarity in biomarkers, likely reflecting ‘physiologically homeostatic’ individuals, provides a basis for future monitoring efforts to assess the impacts of potentially localized threats to health, specifically an increased risk of oil spills, in comparison with GWGUs from unaffected regions.

Values for biomarkers measured in non-breeding GWGUs were largely consistent with published data for this species of GWGUs (Hughes *et al.,* 1993; Newman *et al.,* 1997) and other gull species (e.g. Costantini *et al.,* 2019; Laranjeiro *et al.,* 2020). However, plasma triglycerides levels were relatively low in our population (1.23 ± 0.82 mmol/l) compared with breeding GWGUs in Alaska (9.1 ± 9.9 mmol/l; Newman *et al.,* 1997). Likewise, glucose levels in our study (14.9 ± 3.46 mmol/l) were lower than in breeding GWGUs (17.8 ± 3.3 mmol/l; Newman *et al.,* 1997). This might be because non-breeding GWGUs are experiencing less nutritional stress or lower energy expenditure compared to breeding birds (Totzke *et al.,* 1999; Minias *et al.,* 2019,), or that these physiological markers are upregulated in breeding GWGUs, e.g. due to reproduction itself such as the presence of lipid-rich yolk precursors during egg laying (Williams, 2012). This highlights the importance of considering life-history stage in interpreting physiological ‘health’.

Non-breeding GWGUs displayed sexual dimorphism in mass, with males weighing more than females, as reported for breeding GWGUs in Alaska (Newman *et al.,* 1997). Haemoglobin levels were higher in female GWGUs in the Salish Sea, in contrast to studies of other gulls where either no difference was reported between sexes (Doussang *et al.,* 2015; Indykiewicz *et al.,* 2017) or where haemoglobin was higher in males (Minias *et al.,* 2019). Lighter body mass females in our study had higher plasma triglyceride levels than heavier males, as found in breeding GWGUs and other gulls (e.g. Newman *et al.,* 1997; Indykiewicz *et al.,* 2017), and females also had higher dROMs levels, which have not previously been seen in gulls (Saino *et al.,* 2011; Leclaire *et al.,* 2015). There was no sex difference in haematocrit or OXY. Our results highlight that it is important that any future physiological monitoring of health should take sex into account.

We did not find strong differences in physiological traits, or overall indices of ‘health’ and ‘nutritional state’ using PCA, across the Salish Sea or compared with the west coast of Vancouver Island, in relation to either sampling region or habitat. This is despite the fact that regional differences in anthropogenic activity and degree of urbanization have been clearly demonstrated to impact gull resource use, through both prey quality and availability (e.g. O’Hanlon *et al.,* 2017; O’Hanlon and Nager, 2018). Most GWGUs are resident in the Salish Sea year-round (Hatch *et al.,* 2011) and migrant gulls typically reach non-breeding grounds by November (Hayward and Verbeek, 2020). So, sampling in January and February should have ensured that gulls had overwintered for at least 2 months such that our physiological metrics reflected those of true wintering areas. In addition, data on winter movements of GWGU using GPS tags, from the same study area, show a high level of regional site fidelity with localized home ranges, with capture location being indicative of the wintering region used in >85% of individuals (Hannah Hall, Mark Hipfner, unpub. data).

Haematocrit was one of the few traits to vary among regions, with individuals sampled in the Greater Victoria region having higher haematocrit than individuals in the Northern Salish Sea or Lower Mainland. Though marginally non-significant, birds caught in the Greater Victoria area also had higher dROMs levels compared with birds in the Northern Salish Sea. These data could suggest the potential for some level of poorer health of gulls in the heavily urbanized Greater Victoria area (see Introduction, e.g. if Hct is sufficiently elevated, perhaps due to dehydration, to increase blood viscosity (Bowers *et al.,* 2014)). Haemoglobin was the only biomarker that varied significantly among habitat types (after accounting for other covariates). Though only marginally non-significant, gulls sampled at landfills had higher haemoglobin levels on average than those from urban areas. We also found that body mass was higher in individuals sampled at landfills than natural areas. Higher haemoglobin and body mass suggest landfill use may be associated with better condition in non-breeding GWGUs perhaps due to access to resources since landfill use has been associated with population growth (Duhem *et al.,* 2008), higher body condition (Auman *et al.,* 2008; Steigerwald *et al.,* 2015) and reduced foraging effort (Langley *et al.,* 2021) in some gull species. In contrast, diets reliant on human foods have been reported to have potentially adverse effects on gull physiology (Marteinson and Verreault, 2020). For instance, yellow-legged gulls (*Larus michahellis*) in highly urban environments had poorer nutritional quality with lower levels of omega 3 fatty acids in their plasma (Lopes *et al.,* 2022).

Stable isotope analysis is a useful tool for determining seabird diets over varying time periods (e.g. Hobson *et al.,* 1994) in tissues with known metabolic turnover rates (Hobson and Clark, 1993; Karnovsky *et al.,* 2008). Our data show that birds sampled on the west coast of Vancouver Island, and the Northern Salish Sea, had *δ*^13^C and *δ*^15^N values reflecting relatively more marine input, compared with birds sampled in the Lower Mainland and in associated urban habitats such as landfills (Hobson *et al.,* 1994; Inger and Bearhop, 2008). In a previous study of GWGUs, Davis *et al.* (2017) showed that, during the breeding season, egg homogenate and chick plasma *δ*^13^C and *δ*^15^N values of gulls on the east coast of Vancouver Island, at sites in the northern (Mitlenatch Island) and southern (Mandarte) Salish Sea, indicated primarily near-shore marine dietary sources (e.g. invertebrates such as *Mytilus, Pollicipes and Littorina spp*.) while west coast birds had diets richer in marine fish (Vermeer, 1982; Davis *et al.,* 2015). GWGU colonies in the Salish Sea also had a more varied diet composition than on the west coast of Vancouver Island and Davis *et al.* (2017) suggested this reflected opportunistic foraging on a wider range of potential prey items, including anthropogenic sources (Davis *et al.,* 2015). We also note that baseline potential foods in marine and terrestrial environments can vary tremendously, especially if human foods are involved via landfills (Hobson, 1987; Lato *et al.,* 2021). This complicates comparisons across habitat types and regions unless actual prey types are measured isotopically and considered in any trophic or source estimates. For this reason, we used our stable isotope data in an exploratory sense rather than quantitatively deriving estimates of terrestrial and marine dietary inputs to gulls or their trophic positions. Regardless, even though our stable isotope data suggest clear differences in ecological context (diet, foraging) across regions and habitat types in our study, this was not reflected in physiological signatures of birds using our range of biomarkers.

In summary, our data are most consistent with the idea that most of the gulls we sampled, across different regions and habitat types, were in ‘good’ health with physiological traits in a normal range for physiologically homeostatic individuals. This is the most parsimonious reason why we found few differences in any of the six physiological markers in relation to region and habitat (despite varying levels of urban development and anthropogenic activity) and no covariation with in blood *δ*^13^C and *δ*^15^N values: we sampled ‘physiologically homeostatic’ individuals at all locations and habitats. Our study provides a foundation for long-term physiological monitoring of health of a key indicator species, GWGUs, for which Canada has regional stewardship responsibility, and within the Salish Sea, a biologically important coastal ecosystem that will be increasingly impacted by human activity. Our physiological reference values provide a baseline to compare with future responses to environmental stressors in this species and should enable the rapid detection of emerging threats to GWGU health and population status. Further work combining this physiological approach to health with analyses incorporating age, diet (from stable isotope analysis), contaminant load, infection and habitat use information (from GPS tag studies) will help elucidate the complex environmental and behavioural factors driving variation in health and better understand the impacts of human activities in the Salish Sea ecosystem.

## Supplementary Material

Web_Material_coaf048

## Data Availability

The data underlying this article are available in The Dryad Digital Repository, at https://doi.org/10.5061/dryad.z08kprrrp.
