# Supplementary material for: Physiological health of wintering glaucous-winged gulls in coastal British Columbia
Source: Conserv Physiol. 2025 Jul 7;13(1):coaf048. doi: 10.1093/conphys/coaf048 (PMC12234122; doi:10.1093/conphys/coaf048)

Supplemental Table 1. Fixed effects from the best fit linear mixed effects models, chosen by Akaike Information Criterion for small sample sizes (AICc) for physiological traits, principle components variables, and stable isotopes. Fixed effects in parentheses were not significant covariates and not controlled for in models testing variation by region or habitat. Values in italics/bold = P < 0.05.

	Fixed effect	Estimate	DF
**Body mass** (g)	Sex	***222.1***	1,192
**Triglycerides** (mmol/L)	Sex	***–0.156***	149
**Hemoglobin** (g/dL)	Sex	***–14.98***	3,117
	Mass	***–0.009***	3,117
	Sex*Mass	***0.013***	3,117
**Reactive oxygen metabolites** (dROMs; mg H_2O_2/dL)	Triglycerides	***0.225***	141
**Glucose** (mmol/L)	(Sex)	0.013	143
**Hematocrit** (PCV %)	(Sex)	–0.88	143
**Total antioxidant titres** (OXY; µmol HClO/mL)	(Sex)	–4.06	134
**Principle Component 1**	Sex	**13.8**	1,88
**Principle Component 2**	(Sex)	2.11	1,88
**δ^{13}C ‰**	(Mass)	0.076	1,149
**δ^{15}N ‰**	(Mass)	0.304	1,149

Note: Year was included as a random effect in all models except hemoglobin which was only measured in 2021. Only significant fixed effects were included as terms in subsequent models testing variation of gull mass and physiological biomarkers by a) region or b) habitat. Significant effects of mass or triglycerides were included as covariates whereas sex was included as an interaction term with region and habitat. Glucose, hematocrit, and OXY, PC2, δ^{13}C ‰, and δ^{15}N ‰ did not have any significant fixed effects.

Supplemental Table 2. Principle component (PC) loading scores for physiological variables measured for glaucous-winged gulls measured in southern British Columbia, Canada.

	**PC1**	**PC2**
**Triglycerides** (mmol/L)	0.107	0.645
**Glucose** (mmol/L)	–0.260	0.448
**Hemoglobin** (g/dL)	0.444	–0.104
**Hematocrit** (PCV %)	0.534	–0.382
**Total antioxidant titres** (OXY; µmol HClO/mL)	0.374	0.369
**Reactive oxygen metabolites** (dROMs; mg H_2O_2/dL)	0.564	0.301

Note: We used a subset of data for PCA that included only individuals with measurements for all six biomarkers (n = 90). No samples from 2020 are included in the PCA as hemoglobin was only measured in 2021.

Supplemental Figure 1. Map of human population density/km^2 within the Salish Sea of British Columbia, Canada. Densities are based on 2016 census data and calculated within each census subdivision (Statistics Canada, 2017). Red points are capture locations of adult glaucous-winged gulls in 2020 and 2021.

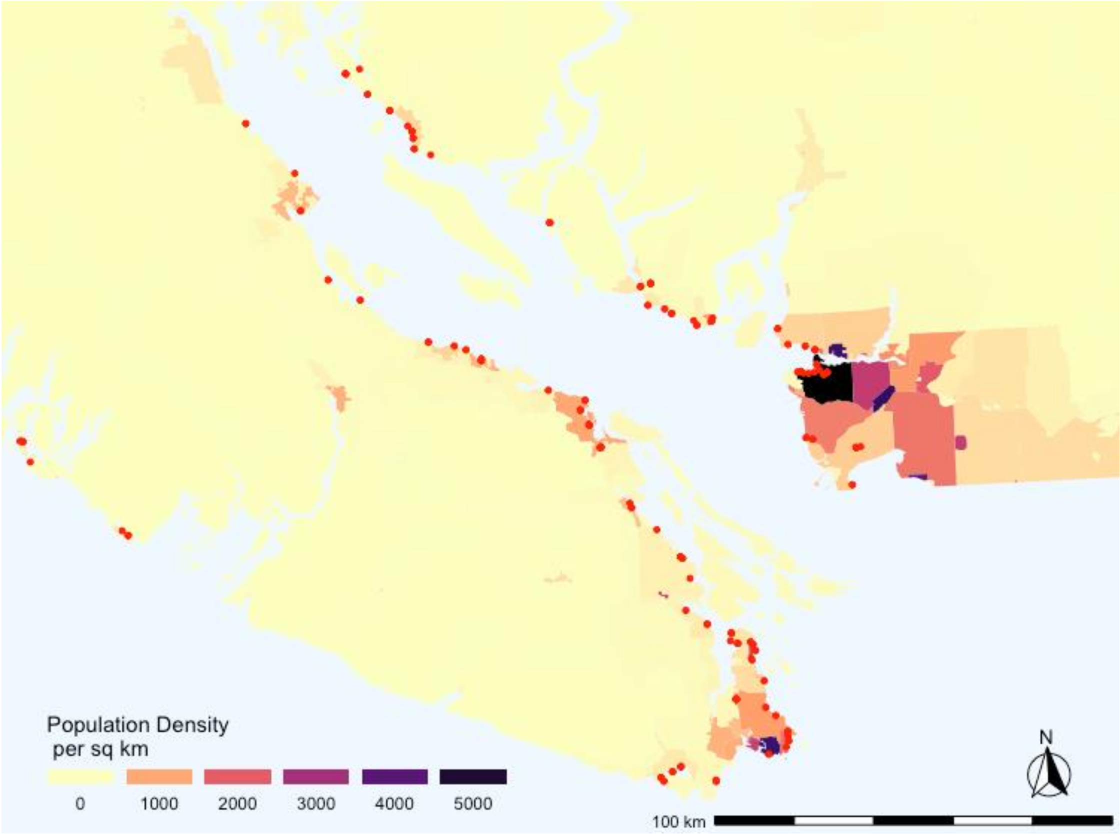

Supplemental Figure 2. Landcover types within the Salish Sea, British Columbia, Canada, displayed at 250 m resolution (North American Land Change Monitoring System, 2021).

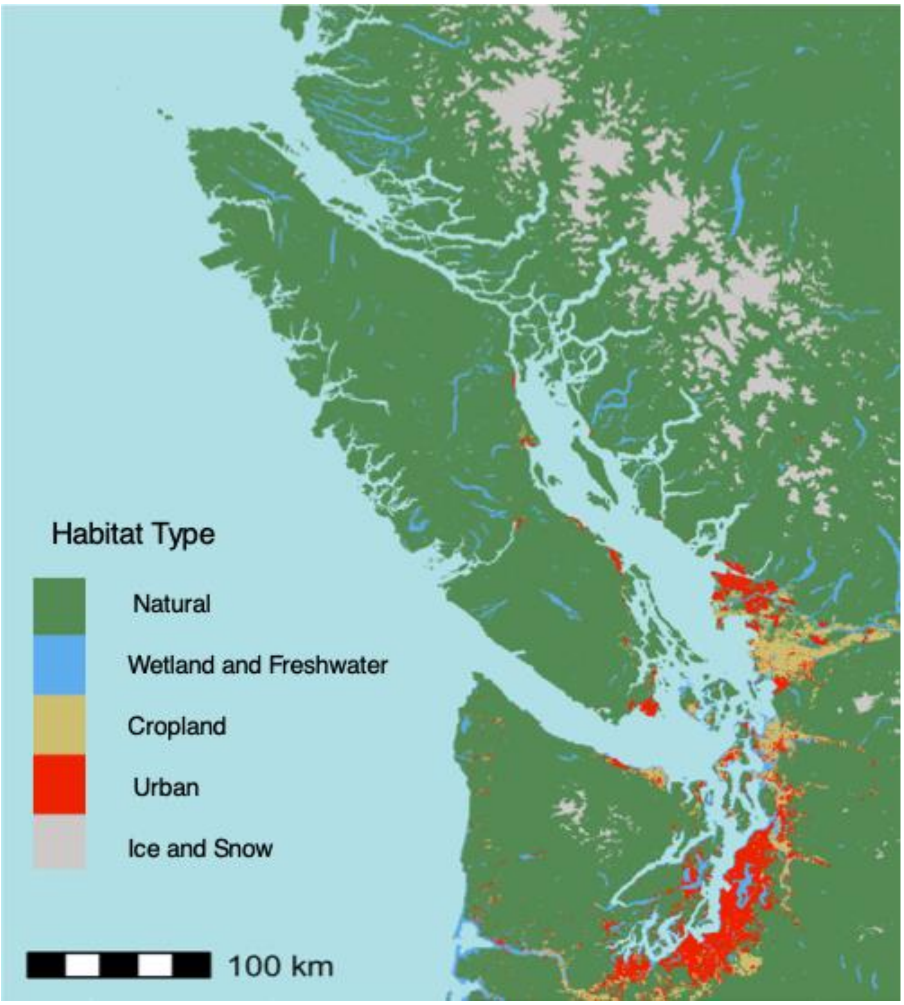

Supplement: Web_Material_coaf048 [file web_material_coaf048.zip › Supp_Tables_Figs_and_Captions.pdf]
